# Recognition of Activities of Daily Living with Egocentric Vision: A Review

**DOI:** 10.3390/s16010072

**Published:** 2016-01-07

**Authors:** Thi-Hoa-Cuc Nguyen, Jean-Christophe Nebel, Francisco Florez-Revuelta

**Affiliations:** Faculty of Science, Engineering and Computing, Kingston University, Kingston upon Thames, London KT1 2EE, UK; j.nebel@kingston.ac.uk (J.-C.N.); f.florez@kingston.ac.uk (F.F.-R.)

**Keywords:** egocentric vision, wearable cameras, ambient assisted living, activity recognition

## Abstract

Video-based recognition of activities of daily living (ADLs) is being used in ambient assisted living systems in order to support the independent living of older people. However, current systems based on cameras located in the environment present a number of problems, such as occlusions and a limited field of view. Recently, wearable cameras have begun to be exploited. This paper presents a review of the state of the art of egocentric vision systems for the recognition of ADLs following a hierarchical structure: motion, action and activity levels, where each level provides higher semantic information and involves a longer time frame. The current egocentric vision literature suggests that ADLs recognition is mainly driven by the objects present in the scene, especially those associated with specific tasks. However, although object-based approaches have proven popular, object recognition remains a challenge due to the intra-class variations found in unconstrained scenarios. As a consequence, the performance of current systems is far from satisfactory.

## 1. Introduction

The number of people aged 65 years or over in Europe and the U.S. will almost double between 2015 and 2060 [1,2]. The Statistical Office of the European Communities (EUROSTAT) projects that, by that year, the ratio between working and retired people will have passed from four-to-one to two-to-one in the EU. In addition, EU Member States spend nowadays approximately a quarter of their GDP on social protection [3]. Such a demographic and economic context raises significant challenges towards health and social care of the older population in terms of increased costs and lack of resources. Ambient assisted living (AAL) systems aim at improving the quality of life and supporting independent and healthy living of older or/and impaired people by using information and communication technologies at home, at the workplace and in public spaces.

AAL environments are embedded with a variety of sensors, either located in the environment or worn by the user, that acquire data about the state of both the environment and the individual and/or allow person-environment interaction. These data are processed using more or less advanced intelligent systems in order to provide services, such as monitoring of activities of daily living (ADLs), prevention and management of chronic conditions, frailty detection and mobility assistance. Many AAL systems aim at detecting and recognizing how people perform ADLs, *i.e.*, tasks that people tend to perform every day, such as eating, bathing and cooking. Knowledge about how a person carries out these activities may be used to detect early signs of dementia [4] or support caregivers’ work [5].

Recently, cameras have begun to be employed in AAL systems to monitor ADLs [6,7], as they provide richer sensory information than the traditional sensors employed in those systems to monitor people, e.g., magnetic sensors, presence sensors and pressure mats. Video-based AAL systems usually employ conventional “third person” vision systems, where the cameras are located in the environment. Therefore, capturing naturally-occurring activities is challenging due to the inherently limited field of view of a fixed camera, the occlusions created by a cluttered environment and the difficulty of keeping all relevant body parts visible, mainly hands, as the torso and head may create occlusions.

An alternative is to mount a camera on the head or the torso of a person and record activities from an egocentric perspective, *i.e.*, from the subject’s own point of view. The development of new wearable camera devices to record videos, such as GoPro^®^ or Google Glass^TM^, makes this possible. As stated by Fathi *et al*. [8], there are three main reasons why the egocentric paradigm is particularly beneficial for analysing activities that involve object manipulation. First, occlusions of manipulated objects tend to be minimized, as the workspace containing the objects is usually visible to the camera. Second, since poses and displacements of manipulated objects are consistent in workspace coordinates, objects tend to be presented at consistent viewing directions with respect to the egocentric camera. Third, actions and objects tend to appear in the centre of the image and are usually in focus, resulting in high quality image measurements for the areas of interest.

**Figure 1:**
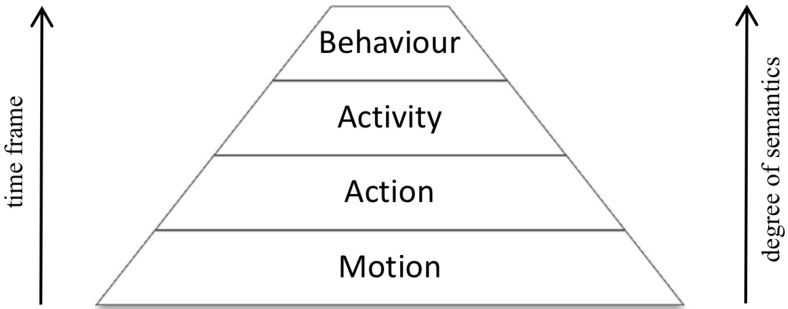
Human behaviour analysis tasks: classification (reprinted from [9]).

Methods for the recognition of ADLs can be classified according to the time frame and the considered degree of semantics. According to this, human behaviour analysis tasks are classified [9] into motion, action, activity and behaviour (Figure 1). At the motion level, events with a time frame of a few frames or seconds are taken into account. This may include saccade (gaze shift) or head motion. Furthermore, tasks, such as object recognition, hand detection, foreground segmentation and gaze estimation, are handled at this level. At the action level, a longer time frame is analysed to recognize simple events, such as open a jar or get water from the tap. At the higher activity level, a sequence of actions in a time frame from several minutes to hours is analysed to recognize activities of daily living, such as preparing a meal, making a coffee or brushing one’s teeth. The difference between an action and an activity is not only about time lapse, but also about a higher semantic level due to more complex interactions between objects and people. For example, a person manipulating an object is performing an action, such as opening a jar, while a sequence of such actions, interacting with different objects, composes an activity, such as preparing breakfast. Although most of the methods follow this pyramidal scheme, some methods analyse activities based on information acquired from the motion level, bypassing the recognition at the action level. These methods usually applied similar techniques to those for action recognition, but considering a longer time lapse.

Approaches applied to egocentric activity recognition can be classified according to the nature of their methodologies. While they could be classified as “low-level” and “high-level”, in this manuscript, the two categories proposed in [10] will be applied, *i.e.*, object-based and motion-based approaches. Motivated by the idea that objects provide essential information about a person’s activity, object-based approaches exploit features from objects, hands and relationships between them to model activities [8,11,12,13,14]. Information about objects and hands, such as their frequency of occurrence, locations and poses, has been used to recognize activities [8]. An activity, such as making coffee, can also be defined as a bag of objects held in the hands, namely coffee, a cup and a kettle [11,12]. Objects that are observed have also been used to recognize actions [12,13,14]. This approach has been proven promising and popular during the last few years. With motion-based approaches, depending on where the cameras are worn, e.g., head, torso or shoulder, camera motion can be exploited to model activities. For example, in [15,16], eye-motion and ego-motion (motion of the camera wearer) were combined to classify indoor office tasks, such as reading or typing, and in [17], the motion of the shoulder-mounted camera was used in order to infer the whole body motion. However, these approaches are only beneficial for activities that require big movements.

The remainder of this paper follows the former hierarchical classification. Section 2, Section 3 and Section 4 present features and methods employed for the recognition of ADLs using egocentric vision at the motion, action and activity levels, respectively. Section 5 summarizes some of the most used datasets in the reviewed works that are available. Finally, some conclusions are given at Section 6.

## 2. Recognition of Elements of Activities at the Motion Level

**Figure 2:**
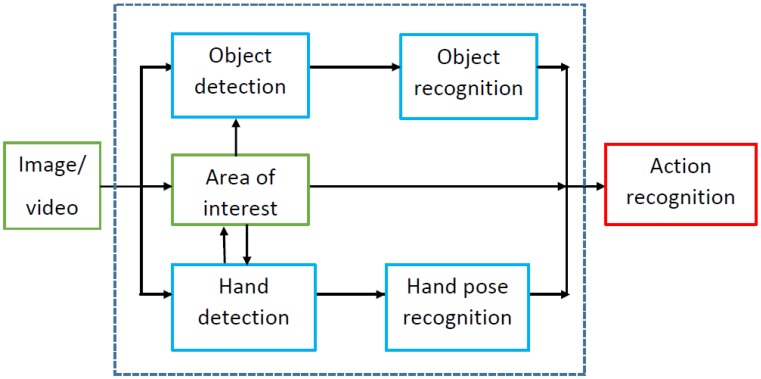
Pipeline for human behaviour analysis at the motion level.

At the motion level, events with a low degree of semantics and a duration of a few frames or seconds are analysed. Most of the object-based approaches follow all or some of the stages shown in Figure 2. Given a sequence of images acquired with an egocentric camera, objects and hands can be detected first. An alternative is to obtain the area of interest, where the activity is taking place and objects are being manipulated. The detection of this area may facilitate the recognition and location of the main relevant objects. Besides, the area of interest may also be estimated after hands are located, as it is usually close to or between them. Information about location and recognition of objects and hands, e.g., frequency of appearance, locations and poses, provides the features to be employed for action recognition at the next level.

### 2.1. Detection of the Area of Interest

The area of interest is the region in the image/video where tasks are carried out. Detection of such an area is important in object-based approaches, as it provides information about where to detect the task-relevant objects in the scene in order to identify ADLs. Foreground subtraction has been proposed to segment the area of interest, as hands and manipulated objects tend to move while people perform tasks [18,19]. These works developed a motion-based approach to segment out manipulated objects and hands by computing dense optical flow and fitting it into multiple affine layers. They proved that foreground segmentation improves the task-relevant object detection. However, the segmentation errors in these works are still high; 48% in [18] and 67% in [19].

The area of interest can also be detected as a region around the focus of attention, *i.e.*, the location where the eyes look, motivated by the idea that people tend to look at the region they are manipulating. One approach to detect the eye gaze is to use an eye tracker [20,21], such as Tobii^®^, or an inward camera [22] to get the eye gaze location with high accuracy.

A different approach to detect the area of interest without using an eye tracker or inward camera is to use saliency information [23,24]. Yamada *et al*. [23] concluded that conventional saliency maps based on colour, intensity, orientation, motion between two images and flicker (absolute difference between the intensity in the current and previous frames) can predict attention in egocentric vision, but performance decreased when applied to videos with large camera motion. Therefore, the estimation of saliency needs to consider ego-motion, *i.e.*, the motion of the camera, to improve the performance.

One traditional method to estimate saliency is to use global contrast [25]. A more popular saliency map estimation method was employed by Walther and Koch [21], in which features of colour, intensity and orientations are used to compute various maps. This method was extended in [12,26] by using ego-motion. Matsuo *et al*. [12] used head motion (camera rotation) to support determining the correct salient object among two objects that have the same saliency. Yamada *et al*. [26] added the camera’s rotation velocity and the direction of movement into saliency maps [21] to improve accuracy. More advanced saliency maps are exploited in [14,27,28,29], where the fusion of multiple information channels, such as motion, spatial contrast and the geometrical centre of an image, is used to estimate the area of interest. More detailed information about saliency maps and associated methods can be found in [30].

**Figure 3:**
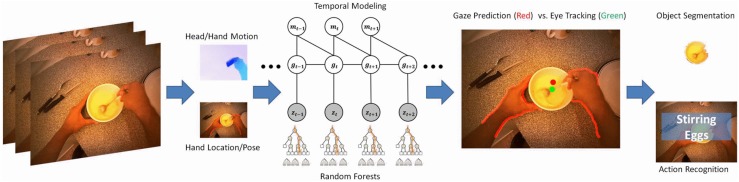
Gaze prediction without reference to saliency or the activity model [32]. Egocentric features, which are head/hand motion and hand location/pose, are leveraged to predict gaze. A model that takes account of eye-hand and eye-head coordination, combined with temporal dynamics of gaze, is designed for gaze prediction. Only egocentric videos have been used, and the performance is compared to the ground truth acquired with an eye tracker (reprinted from [32]).

An alternative idea has been to take advantage of the hands’ positions, since they tend to inform about the area of interest. Buso *et al*. [31] use hand segmentation to infer their positions and orientations to build a top-down saliency model, which is able to estimate observer’s expectations, whereas Pirsiavash and Ramanan [11] combine spatial features near the centre of the images and skin detection to predict gaze. Another approach was proposed by Li *et al*. [32] using head/hand motion and hand location/pose to predict the gaze without reference to saliency or the activity model, achieving an area under the curve (AUC) score of 87.8%. Their system extracts features that contain the manipulation point, the global motion, the hand motion and the hand configuration, as shown in Figure 3.

Foreground subtraction improves significantly active object detection even when segmentation is flawed. However, it generally cannot deal with static objects, such as a coffee machine. This limitation can be addressed by gaze information, which has been shown to improve action recognition. This is particularly important, since progress in gaze prediction suggests that eye-trackers are no longer necessary. Although usage of visual cues (spatial-temporal-geometry saliency maps) is based on sound principles, its added value in terms of object recognition has remained limited. Recent work by Buso *et al*. [31] demonstrates that the introduction of semantics in the computation of the area of interest has the potential to significantly improve performance. However, the challenge shifts to accurate hand and object segmentation.

### 2.2. Object Detection and Recognition

Objects in egocentric videos are important elements in many activity recognition systems, as they may provide essential information about a person’s activity [8,11,12,13]. Relevant objects to recognize ADLs with egocentric vision can be grouped into four non-exclusive categories:

- Salient/non-salient objects: objects in egocentric videos that are fixated, *i.e.*, focused on by the user’s gaze or not;
- Manipulated/non-manipulated objects: objects in egocentric videos that are in hands or not;
- Active/passive objects: objects that are relevant to tasks, *i.e.*, salient or manipulated, or not.
- Multi-state objects: objects that have changes in terms of colour or shape.

An action may be defined as a bag of active objects, while passive objects may contribute with some contextual information to action classification [11,12]. For example, making tea can be represented as a bag of objects, such as a tea bag, a cup and a spoon. Object recognition in first-person videos shares some challenges with other video-based applications, such as background clutter or a real-time processing requirement. It also has its own challenges, as partial occlusions between hands and manipulated objects, strong ego-motion and dramatic viewpoint changes due to object manipulation. To tackle the background clutter problem, some authors proposed detecting the area of interest in the scene before recognizing objects [8,13]. So far, objects in egocentric settings have been investigated at different levels, from detecting active objects without classifying them into categories [33,34,35,36] to recognizing all of the objects in the scene [13,37] or recognizing only active objects [11,12,18,19,28,38]. This section provides a review of object detection and recognition approaches in egocentric videos for the recognition of ADLs.

Detection of active objects is crucial for object-based activity recognition approaches, and there are several proposals for this task. Feature point descriptors, such as Scale-invariant Feature Transform (SIFT) [39], Speeded Up Robust Features (SURF) [40] and Histogram of Oriented Gradients (HOG) [41], are widely used to detect objects. SIFT is a gradient-based algorithm that detects and describes local features in images; it is invariant to image translation, scaling and rotation. Therefore, it has been largely used [18,19,38,42] for object detection, as objects tend to present different positions, scales and orientations in egocentric settings. However, Fathi *et al*. [18] proved that a merely standard SIFT-based system will be sensitive to background clutter and hand occlusions. To handle these problems, the area of interest needs to be detected and/or segmented first. Approaches that use SIFT on a segmented foreground to detect active objects in the scene were proposed in [18,19,42]. Despite SIFT’s accuracy, an alternative detector/descriptor, SURF, which uses Hessian matrix approximation for key point detection and is based on the sum of Haar wavelet responses for key point description, has been very attractive due to its low processing cost. Moreover, SURF was shown to provide the same performance as SIFT [43]. With egocentric vision, SURF descriptors applied to dense sampling were combined with saliency information as a weighted value of the area of interest to detect active objects in the scene [14,27].

**Figure 4:**
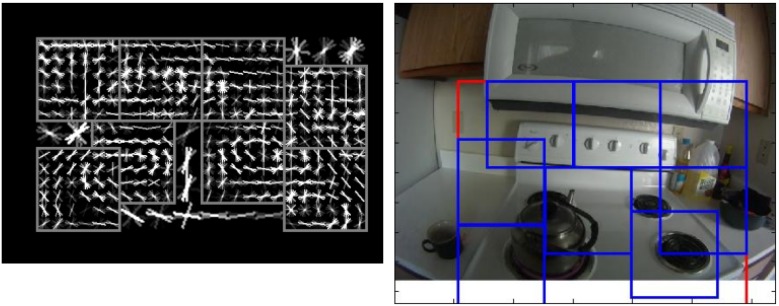
A part-based object model for a stove in an activities of daily living (ADLs) dataset using a HOG descriptor (reprinted and adapted from [11]).

A similar gradient-based descriptor, HOG, which is computed on a dense grid of uniformly-spaced cells, was used in a part-based object detection model [44,45] and has been widely applied [11,12,19,46] for detecting objects. This model represents an object as a mixture of multiple deformable parts. This is especially appropriate for multi-state objects in ADLs, which may change their appearance regularly during manipulation, e.g., an open fridge looks different from a closed fridge. Figure 4 shows a model for a stove. Particularly, this model has been combined with temporal integration and background segmentation to detect active objects [19], with spatial features and a skin detector [11] and with gaze estimation [12]. Tomas McCandless [46] used this part-based object model to detect objects, which then provide information for action recognition. These methods allow for localization of objects in the scene, while saliency-weighted signatures are used to only recognize the presence of an object without localizing it. HOG was shown to outperform the SIFT-based system for object recognition [19].

As an alternative to gradient-based descriptors, colour and texture histograms, which may provide important information for object recognition [47,48], were used within a super-pixel approach [13,18]. In this method, texture descriptors are computed and quantized into 256 classes, which are built by K-means clustering. Ten colour descriptors are extracted and quantized into 128 classes. However, this colour-based method is sensitive to variations in illumination and fails to distinguish objects that co-occurred, e.g., they failed to classify water and cup (a cup of water) or ketchup and chocolate (when both spread on bread).

**Table 1:** Combination of features and machine learning methods for object recognition with egocentric vision. GTEA, Georgia Tech Egocentric Activities.

Target	Paper Year	Approach	Dataset ${}^{1}$	Results
	[38] 2009	Standard SIFT + multi-class SVM	Intel 42 objects	12%
	[19] 2010	Background segmentation + temporal integration + SIFT, HOG + SVM	Intel 42 objects	86%
	[18] 2011	Background segmentation + multiple instance learning + transductive SVM [49]	GTEA	in a table, but stated 35% according to [28]
Active objects (manipulated or observed)	[13] 2012	Background segmentation + colour and texture histogram + SVM super-pixel classifier	GTEA Gaze, GTEA Gaze+	n/a
	[11] 2012	Part-based object detector (latent SVM) on active images + spatial + skin detector	ADL	n/a
	[12] 2014	Part-based object detector (latent SVM) + “salient” assignment based on estimated gaze point	ADL	n/a
	[28] 2014, [14] 2013	Visual attention maps (spatial + geometry + temporal) combined with SURF + BoVW+ SVM	GTEA, GTEA Gaze, ADL	36.8% 12%
	[27] 2014		Their own dataset	50%
General objects (All objects in the scene)	[11] 2012	Part-based object detector (2010) + latent SVM	ADL	19.9% fridge to 69% TV

${}^{1}$ See Section 5 for details.

So far, the part-based object detection model based on HOG [44,45] has been shown to be the most popular model for object recognition in ADLs with egocentric vision due to the fact that objects in ADLs regularly change their appearance during interactions.

After detection, active objects must be recognized to provide information about which objects are manipulated in the current actions. Support vector machines (SVM) have been shown to be the most used tool for training and recognizing objects [11,12,13,18,19,27,28,38], as shown in Table 1, due to their capability to deal with problems that are non-separate, non-linear and multi-label, using low computational resources. However, as SVM requires many annotated training data, another approach, weakly supervised learning, where limited annotated data are used for training, has emerged. Particularly, in [18], multiple instance learning was used to match object instances across frames in order to locate objects. Table 1 shows the combination of computer vision and machine learning methods for object recognition.

Despite many efforts to recognize objects in egocentric videos, it is still far from being solved. Recognition of similar objects, such as coffee and tea, or small objects, such as dental floss, or the co-occurrence of objects has not yet achieved good results. Moreover, object recognition in unconstrained environments where training and evaluation are performed in different settings remains a challenge to researchers. Therefore, new object representations that are robust to intra-class variation are needed to provide higher accuracy for object recognition.

### 2.3. Hand Detection and Recognition

Hand detection and segmentation in egocentric videos are gaining more and more attention as they have been recognized as critical for understanding activities requiring hand object manipulation, *i.e.*, making coffee, or analysing hand-eye coordination [8,21,50,51]. Hands and arms in egocentric videos have been studied at different levels, from detection, localization, segmentation [50,52,53,54,55] to gesture recognition [52,56] and disambiguation between those of camera wearers and those of their potential partners [57].

**Figure 5:**
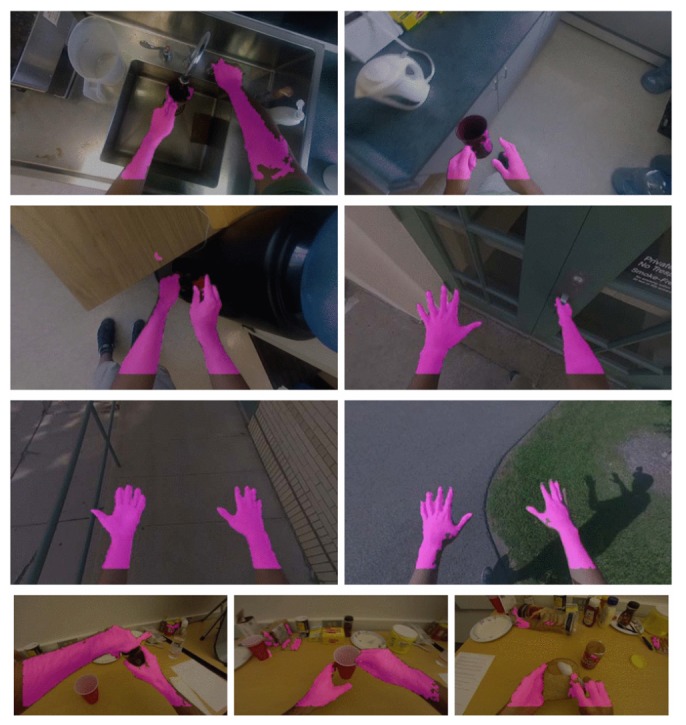
Pixel-level hand detection under varying illumination and hand poses (reprinted from [50]).

As stated in the survey by Morerio *et al*. [58], colour is a simple, but good feature for detecting the location of hands, particularly if a proper colour space is considered, such as Lab, HSV [59] and YCbCr [60]. This feature is usually combined with others, such as texture and contours [50,53]. However, colour features are sensitive to variations in illumination and shadows. This problem was partially addressed in [50], where a pixel-level hand detection and segmentation was proposed, obtaining good results. Li and Kitani examined the local colour information, *i.e.*, the colour of pixels surrounding the evaluated pixel, and showed that using a small patch improves results. This confirms their idea that the information surrounding the pixel of evaluation should help to discriminate hand regions. Moreover, their experiments showed that hand segmentation under various illumination conditions can be obtained by using a spare 50-dimensional combination of colour, texture and gradient histogram features, *i.e.*, SIFT and HOG. They tested the method on their own dataset with 200 million labelled pixels and a public egocentric indoors dataset (Georgia Tech Egocentric Activities (GTEA)) and obtained close to 80% accuracy. Figure 5 shows some of their results.

Betancourt *et al*. [53] proposed a two-level sequential classifier for hand segmentation. They proposed different combinations of features and classifiers as hand detectors, namely colour histograms, HOG and GIST [61], a global descriptor based on colour, intensity, and orientation, and as machine learning methods, they tested SVM, decision tree and random forest. They evaluated their approach on their own dataset of 2835 video frames. They found that the combination HOG-SVM yields the best performance for detection (90% true positive and 93% true negative). Despite the presented approaches, hand segmentation is still far from being solved due to computational cost and variations in illumination [10]. A wider overview of hand detection can be found in [58].

Gesture recognition or hand pose detection in egocentric videos plays a fundamental role, as it provides valuable information for action classification, such as grabbing a cup or pouring water into a cup. Gesture recognition has been proven to be enhanced by using segmented images of hands instead of full frames during the test phase [52]. Although this work is not focused on the recognition of gestures in ADLs, it shows the advantage of pre-segmenting the hands. Exemplar SVMs were used for testing the method on their own dataset, where only five symbolic gestures had to be recognized. Another approach for hand pose detection from an egocentric viewpoint is proposed in [56] exploiting the depth information acquired with a chest-mounted RGB-D camera. That work showed that the depth cue provides an extraordinarily helpful signal for pose estimation in a first person perspective. A wider overview of hand gesture recognition systems can be found in [62].

Although hand segmentation has achieved reasonable performance, reliance on colour information makes the classification of objects that have a similar colour as hands difficult. In addition to hand segmentation, gesture recognition and hand gestures need to be investigated further, so that they can be exploited for ADLs.

## 3. Recognition of Elementary Actions

The outcome of the completion of the motion level is the detection and recognition of objects and hands. This section reviews works and techniques for the recognition of basic actions, such as closing a jar, taking an egg or opening a lid.

The bag-of-words (BoW) approach has been shown to be a popular method to model actions and has been extended in different ways: bag of active objects [20,63,64], bag of object (and wrist) interactions [65] and bag of oriented pairwise relations [66]. Particularly, an action can be modelled as a bag of active objects to describe the object used in the scene [20,63,64]. There, a histogram representing the occurrences of objects is used as the feature. In addition, modelling of actions, such as pick up hammer, was performed using a bag of interactions, including three types of interactions: object-object, object-wrist and between body parts [65]. In this approach, the wrist is detected using a visual marker, which is treated as an object. Vocabularies are built from spatio-temporal relations of objects and body parts. Two histograms of such relations are used to represent actions. Another BoW approach is proposed in [66], where spatial pairwise relationships between SURF points detected in an image are used to build the codebook. In their datasets, actions like remove cover or pick bottle were recognized, obtaining an average frame classification accuracy of 36.5% on the GTEA and Leeds datasets, which is better than the simple bag of SURF words (28.8%) on the GTEA dataset.

Object-to-object and hand-to-hand relationships were exploited in [8,63]. Manipulated objects (objects in hands) were first defined and investigated in [8] to recognize activities performed in a kitchen. Fathi *et al*. proposed a graph-based approach to represent ADLs by actions, objects, hands and interaction between them. First, they segmented the foreground from the background. The foreground contains hands and manipulated objects. Then, they used features obtained from objects, hands, interactions between objects and those between hands to model actions. This approach was also applied to the GTEA dataset, obtaining a success rate of 32.4% on 64 action classes.

**Figure 6:**
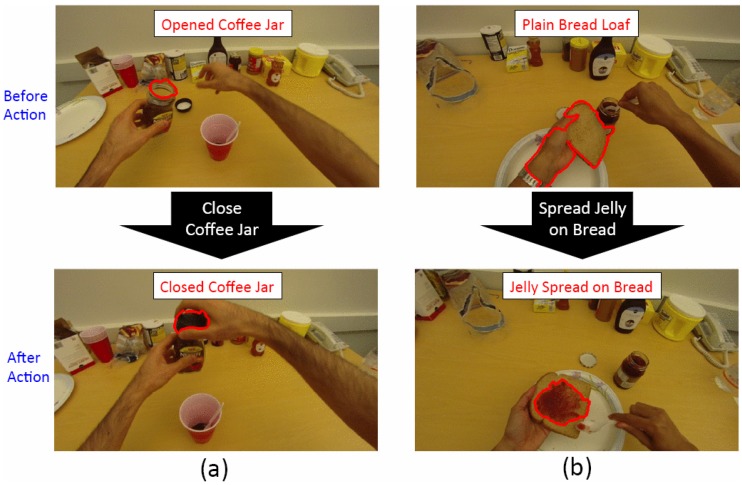
Action recognition based on changes in the state of objects (reprinted from [64]).

Changes in the state of objects have also been analysed to support action recognition, motivated by the idea that objects may change states while a task is being performed. Fathi and Rehg [64] proposed a new approach to model actions in egocentric videos based on the changes they make in the objects, as illustrated in Figure 6. This method achieved a 39.7% action recognition accuracy (based on 64 action classes) and outperformed their previous work [8] (32.4%) using the same GTEA dataset.

Actions can also be modelled using a global descriptor, such as GIST [61], which provides a rough description of the scene based on the colour, intensity and orientation of the scene [67]. Since that method achieved only a classification accuracy of 9.38% on 29 classes, this suggests that GIST is not a suitable descriptor for ADLs recognition.

**Figure 7:**
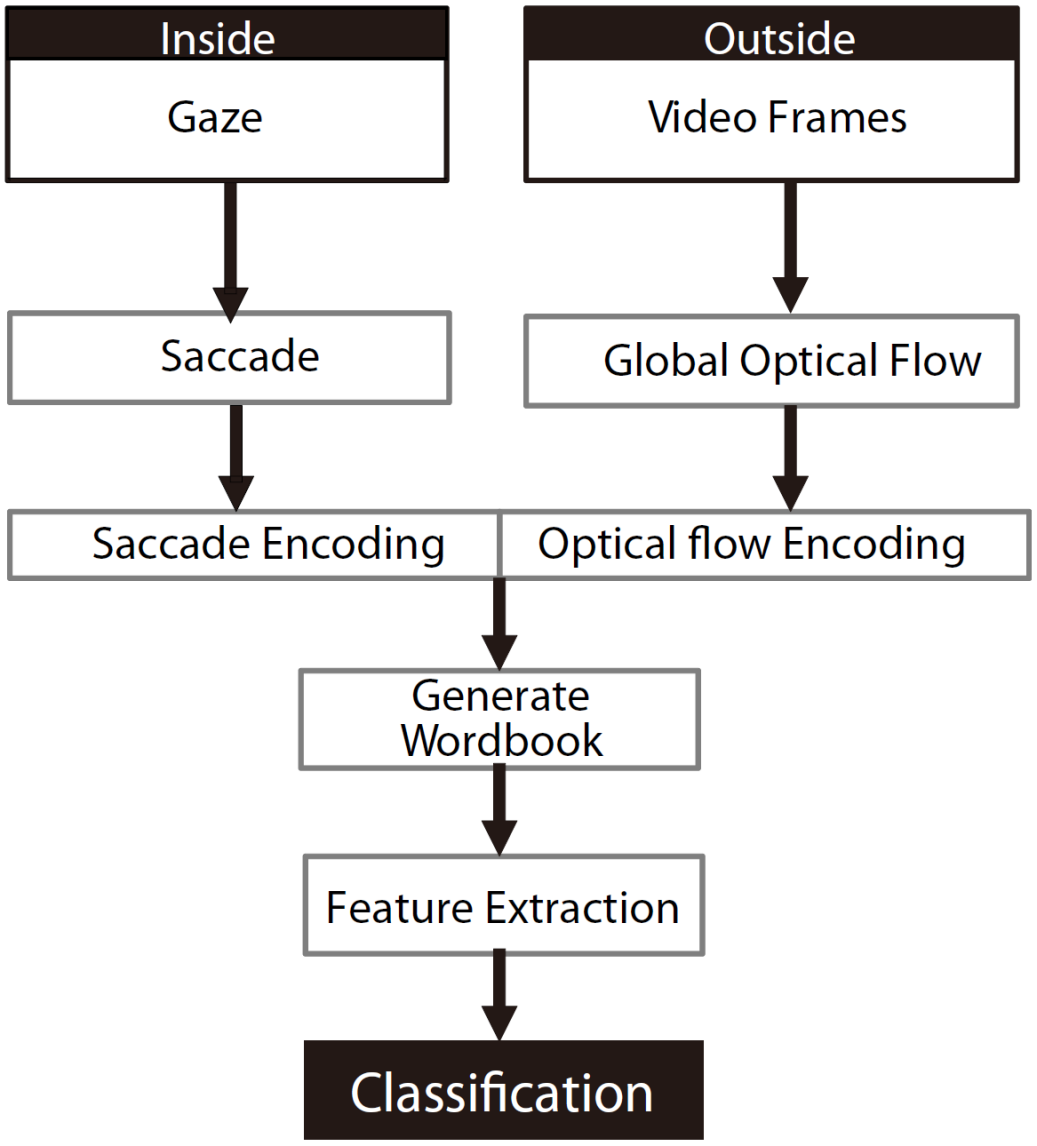
Flow chart for indoor office task classification (reprinted from [15]).

Besides object-based approaches, there are motion-based approaches that exploit hand positions [20,68], eye motion [15,16] and ego-motion [15] to model actions. Hand positions were used to model five actions, namely picking up, placing, lining up, stapling and folding in [20,68]. That system obtained a recognition accuracy of 91.6%. Eye motion was also exploited for recognizing actions in [16] (Figure 7). Shiga *et al*. [16] evaluated their approach on their own dataset, which consists of six office tasks, such as watch a video and write text, and achieved a high accuracy of about 90%. Another approach is the combination of eye-motion and ego-motion, which was used to improve the classification accuracy for indoor office tasks, such as reading, typing, browsing and writing in [15], obtaining a mean average precision of 57%. However, this method is limited to office tasks that involve big eye and head movements.

After feature extraction, machine learning methods come into play in order to learn and classify actions. k-NN was used for action recognition in [67] employing GIST features. Spriggs *et al*. explored 1-NN and 3-NN for classification, using the Euclidean distance, obtaining an average frame classification performance of 48.64% on 29 classes, such as take oil and crack egg. However, since their dataset was not large, their results are not conclusive. Similarly to the motion level, support vector machines are the most used classifiers for action recognition. Fathi *et al*. [13] used an SVM classifier to learn actions, such as scoop jam using a knife and close a jar, with object-based and gaze location appearance. They also incorporated future manipulation features, as the gaze is usually ahead of the hands in the hand-eye coordination while performing tasks. Particularly, if the gaze point (eye gaze) in the image is provided, accuracy on action classification reached 47% (compared to 27% in [8] on the same dataset). Besides, a linear SVM was used in [64] to train a classifier for actions such as open a jar based on the state changes of the manipulated objects. Moreover, a multi-class SVM classifier was used to learn histograms of oriented pairwise relations in order to recognize actions like remove cover and pick bottle in the GTEA and Leeds dataset in [66], getting an average frame classification accuracy of 36.5%.

In motion-based approaches, where the motion of eyes and head is considered, SVM has also been used with different approaches: multi-class non-linear SVM [16], multi-channel SVM [69] and linear SVM [15,70]. Ogaki *et al*. [15] used linear SVMs to train their classifier on features extracted from gaze motion to classify actions, such as write and type. Particularly, they trained a one-*versus*-all SVM to evaluate each class’s performance and a multi-class SVM to evaluate confusion between classes. Similarly, in [70], to classify actions, such as typing, a linear SVM was selected using a Fisher vector as the feature encoder, and a non-linear SVM classifier was trained on bag-of-word feature vectors. Besides, in order to combine both global and local motion features, Ryoo and Matthies [69] applied multi-channel kernel SVM for recognizing actions, such as hand shake and wave. In [16], two multi-class SVMs were trained and then fused to recognize activities, such as typing. Particularly, a multi-class SVM was trained on visual features, which are extracted from the local image around the gaze region, and a multi-class SVM was trained using extracted gaze motion features, which are obtained from an inward eye camera. However, since these motion-based approaches only exploit motion, but not information about the objects, they are not appropriate for activities involving the manipulation of objects, which are the most common in ADLs.

Hidden Markov models (HMMs) have been a popular learning method for action recognition due to their excellent adaptability to problems that have a significant time variation, as happens in action recognition. Simple actions, like picking up, folding and placing, were recognized in [68] using traditional HMMs. This method was compared with k-NN in [67]: with a supervised HMM using GIST features, the system achieved classification accuracy of 9.38% on 29 classes, such as take oil and crack egg, which is much lower than k-NN, which got an average frame classification performance of 48.64%. Contrarily, Spriggs *et al*. found that using a simple k-NN model outperforms the standard HMM for high dimensional data.

Another learning algorithm used is AdaBoost, where the importance of samples that are misclassified in an iteration is increased for the next iterations. Particularly, Fathi *et al*. [8] learned binary action classifiers for recognizing 64 action classes, like pouring water, using AdaBoost after concatenating features of segmented objects and hands to build the feature vector. The method used 200 iterations of the AdaBoost algorithm for every frame on those features and obtained an accuracy of 32.4% on 64 action classes on the GTEA dataset.

Temporal templates, which are single images that incorporate motion history in a sequence, were used in [71] for characterizing actions like remove a lid. Then, these templates are classified using image matching techniques. Sundaram and Cuevas used three matching techniques and found that normalized cross-correlation performed the best. Particularly, templates of hand motions over time for each action were exploited to classify actions, leading to an accuracy of 60.99% for action recognition with 12 classes.

Using unsupervised learning, multi-task clustering, *i.e.*, learning multiple tasks simultaneously, has been demonstrated to give better results for action recognition in egocentric vision with respect to traditional single task approaches [72]. Moreover, motivated by the idea that people tend to perform the same actions in the same environment, Yan *et al*. proposed a multi-task clustering framework by looking at tasks performed by multiple users simultaneously.

Although multiple approaches have been proposed, the performance is still largely unsatisfactory when dealing with a large number of action classes, as ADL requires. In particular, intra-class variation in such unconstrained environments makes action recognition extremely challenging. Object-based methodologies, especially those associating tasks with objects, *i.e.*, frequency of appearance, state changes and interaction with hands, have proven the most promising. However, since motion-based features are also often informative, it is likely that a robust action recognition system will require combining those two types of approaches.

## 4. Recognition of Complex Activities

At this level, tasks, such as preparing a cake, preparing rice, making coffee, making bed, cooking and washing hands, are analysed and recognized. These activities usually consist of a sequence of actions in a particular order at a higher degree of semantics and require minutes to hours to be carried out. It is worth noting that not all approaches for activity recognition follow the scheme presented in Figure 1. Some methods may recognize activities directly from information about objects, hands and the context obtained at the motion level. Particularly, models applied for activity recognition can be grouped into five non-exclusive categories depending on how an activity is represented:

- Activity is a combination of actions [8,71];
- Activity is a combination of active objects [11,12,42,46,63];
- Activity is a combination of active objects and locations [14,71];
- Activity is a combination of active objects and hand movements [20];
- Activity is modelled by other information, *i.e.*, by the eye gaze region [73], motion information [74], colour features [75], ego-motion, image localization, colour and spatial information [17,76].

Similarly to action recognition, bag of visual words is shown to be a popular method across works on activity recognition, but at a higher semantic level. Bags of active objects with a corresponding number of occurrences and the importance of objects were used in [63] to describe activities, such as making a cake. This method was applied in an immersive virtual reality environment, obtaining a recognition precision of 89% at the activity level and 76% at the action level for 10 everyday home activities. Another extension of BoW was proposed by Sundaram and Cuevas [71], where activities were represented as bags of actions. Actions are fixed sequences of interactions, which were defined by verbs, object and location. For example, making a cup of coffee contained a bag of actions, such as pouring water from a jug into a cup, which included two interactions, pour water from the jug and place the jug back in its original position. This method was validated on five activities performed in a kitchen. The method is sensitive to noise, and the accuracy decreases significantly when actions from another activity class take place.

**Figure 8:**
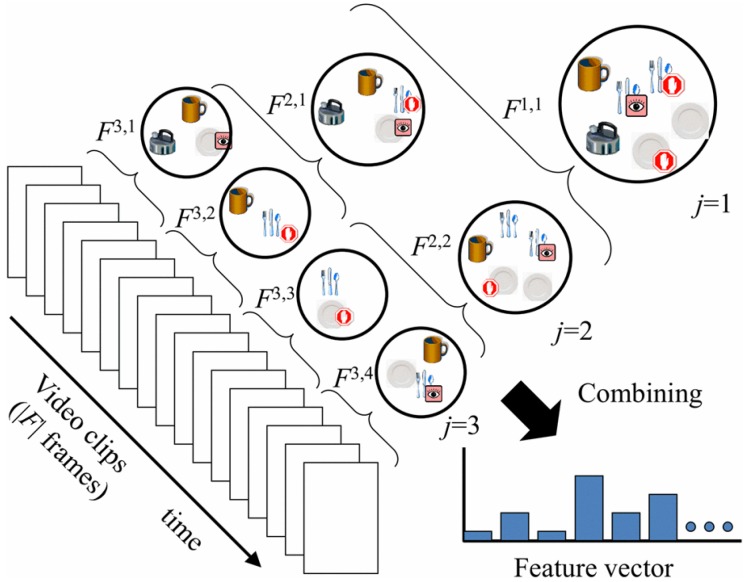
Temporal pyramid representation of a video sequence (reprinted from [12]).

**Figure 9:**
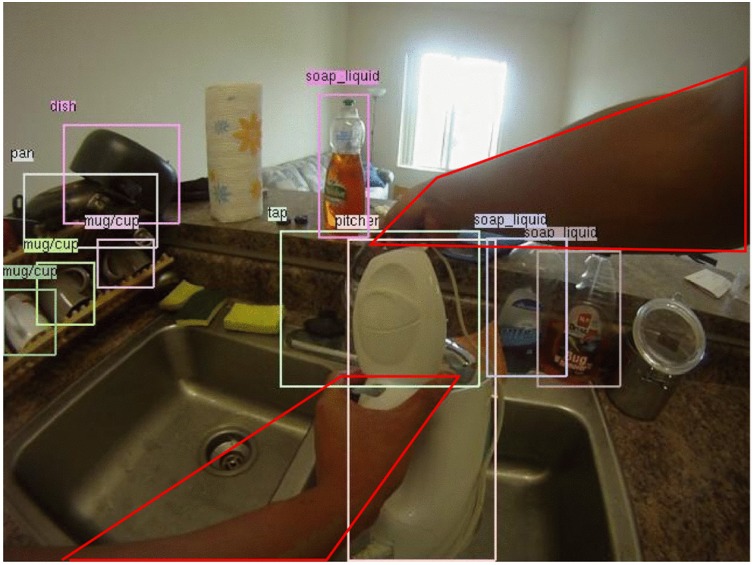
Detection of hands and objects in the ADL dataset (reprinted from [11]).

An extension to the bag-of-words approach is the use of space and time pyramids [11,12,46]. Pirsiavash and Ramanan [11] proposed an activity representation based on temporal pyramids to describe object use over time. They collected a large and fully-annotated dataset, the ADL dataset, with 18 daily indoor activities, such as combing hair or watching TV. Objects in this dataset were labelled as “active” (in hands) or not. For every frame of a given activity, they used a part-based object model [11] to record a score based on the most likely position and scale for each of their 42 object classes. Averaging this score over all activity frames yielded a histogram of object scores for a specific activity. The method went on to temporally split the video into halves in a pyramid fashion, each time calculating the object score histogram and, thus, ending up with an activity model that describes object use over time. A linear SVM was trained on these models. Trained with all the objects, the system achieved a 32.6% activity classification accuracy; however, trained with only active objects, it reached a 40.6% accuracy. With idealized perfect active object detectors, the performance was increased to 77%. Similar results were obtained by [12], where temporal pyramids were extended to include objects to which people pay attention, as shown in Figure 8. Objects were non-exclusively labelled as salient/non-salient and manipulated/non-manipulated. Another extension of this model, which improved the performance further, was introduced in [14] exploiting the information about the location where the activity takes place. This was motivated by the idea that an activity in egocentric videos can be defined as a sequence of interacted objects inside a particular room. For example, cooking cannot be performed in a living room, while cleaning the house might require a user to move around various places. Their object recognition approach was based on saliency information, and the location recognition system used a global image descriptor and a linear SVM. Their activity recognition uses temporal pyramids in an extended way that represents activities as sequences of active objects and places. These methods confirm that ADL recognition is mainly about the objects, especially the ones being interacted with. However, a limitation of all of those temporal pyramid-based methods is that their scene descriptors confuse functionally-similar activities, such as brushing teeth and dental floss. Figure 9 shows a frame from the activity making a cup of coffee in their dataset in which hands are detected and objects are located and labelled.

In [46], the temporal pyramids approach [11] was extended with object-centric spatial pyramids. Tomas McCandless proposed a boosting approach that automatically selects a set of efficient spatio-temporal pyramids among a randomized pool of candidate partitions to create a strong classifier. First, they ran the object detectors (both active and passive objects). Then, they constructed a series of candidate space-time pyramids that have object-centric cuts, *i.e.*, sampling of bin boundaries is carried out near those objects that are active (those being manipulated by the user, such as an open fridge). Last, they used multi-class AdaBoost and obtained a classification accuracy of 38.7% on the ADL dataset. They showed that object-centric cuts improved the result. However, this result is still low, and training takes longer.

Another extension of BoW is the Fisher vector [77] (FV), which goes beyond counting the occurrences of visual words to encoding much richer information of the low level feature distribution in the image. It was used in [74] to encode the features after several descriptors (HOG, histograms of optical flow (HOF) and motion boundary histograms (MBH)) were computed. HOG represents static information, while HOF and MBH describe motion information. The system recognized activities, such as watch TV, write and eat, with an accuracy of activity recognition of around 80% on their LENA dataset, which mainly focuses on non-manipulation activities recorded with a Google Glass^TM^ device. This approach had been applied for third-person action recognition before in [51].

**Figure 10:**
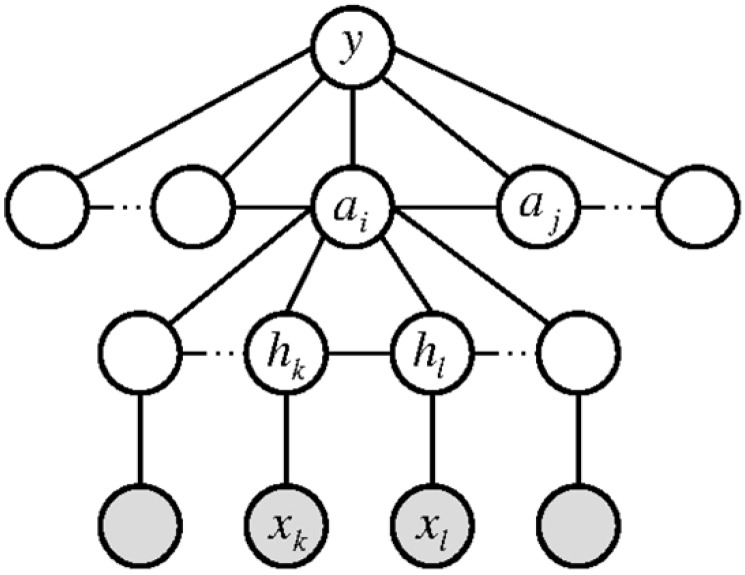
Graph-based framework’s model. An activity *y* is a sequence of actions $a_{i}$, and each action is represented by objects and hands. During testing, objects and hand labels $h_{k}$ are assigned to regions $x_{k}$ (reprinted from [8]).

Activities can also be represented as graphs [8], as illustrated in Figure 10. Fathi *et al*. proposed a graph-based approach to represent activities of daily living by actions, objects, hands and interactions between them. Manipulated objects are investigated to recognize activities performed in a kitchen. They used features that capture the information about objects and hands, namely frequency, optical flow, locations, poses, the relative location among objects, the left/right hand relative location relationship and the hand to hand relationship. Then, the method learned action classifiers using 200 iterations of AdaBoost for every frame. To recognize activities from the classified actions, they used AdaBoost with 10 iterations on the histogram of action frequencies for each sequence. The system could recognize six out of seven activities correctly, obtaining a frame classification accuracy of 45%. A particularly interesting point is that the incorporation of hand location and hand pose provided the best performance, which suggests that information about hands improves action recognition.

**Figure 11:**
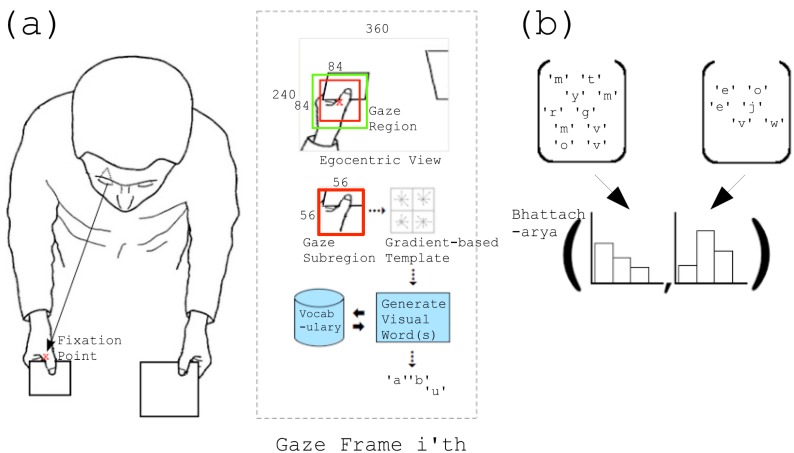
Visual explanation of the proposed method. The region around the fixation point is extracted and encoded using a gradient-based template. These templates are used to build the vocabulary, which is then applied to generate a BoW representation for an activity (reprinted from [73]).

Unlike most of the approaches that use objects to model activities, Hipiny and Mayol-Cuevas [73] used gaze information to model 11 activities, such as making a cup of tea, from about 200 egocentric videos along with data acquired with the ASL Mobile Eyegaze tracker. Gaze regions were analysed using BoW with a weighted multiple voting scheme. A gradient-based template, the Dominant Orientation Templates [78], was used to encode each gaze sub-region. Figure 11 describes this method. The accuracy is 59% on 11 classes of activities, such as cook noddles or make a cup of tea.

Ego-motion was considered by Karaman *et al*. [17,76], which combined several features to analyse activities from shoulder-mounted camera videos for the monitoring of patients with dementia. They used the camera motion detection method to estimate the global motion, e.g., ego-motion, such as sitting or standing. Regarding the features, they used cut histograms to characterize the dynamics of activities, translation parameter histograms to indicate the strength of ego-motion, a BoW approach with SURF features for image localization and an MPEG-7 colour layout descriptor to express colour and spatial information in images. Each activity was then defined as a hierarchical HMM, in order to contain multiple states of the activity. They tested their approach on their own dataset, which included seven different activities, such as making coffee, and achieved a recognition accuracy of 67%. Yu and Ballard also took advantage of the HMM framework [20] for recognizing activities, e.g., unscrewing a jar, stapling a letter and pouring water. Instead of traditional HMMs as in their previous work [68], they used parallel HMMs (PaHMMs) consisting of two sets of HMMs to model hand movements and object sequences, *i.e.*, the sequence of fixated objects by the eye using an eye tracker. The probability estimates of the two models were then combined for recognizing the actions. The system achieved an accuracy of 96.3% with three actions.

While HMMs have been used widely at the action level, dynamic Bayesian networks (DBNs) have been applied widely at the activity level. A DBN is an extension of hidden Markov models in that a “DBN can have multiple hidden and observed states which have complex interdependencies” [79]. A three-level DBN was employed to infer locations, objects and activities from a sequence of actions in [71]. Only egocentric videos from a shoulder-worn camera are used for recognizing five activities performed in a kitchen, like washing a dish, based on object use and the current context (location). However, the proposed model is sensitive to noise and the order in which the manipulations occur within an activity.

**Figure 12:**
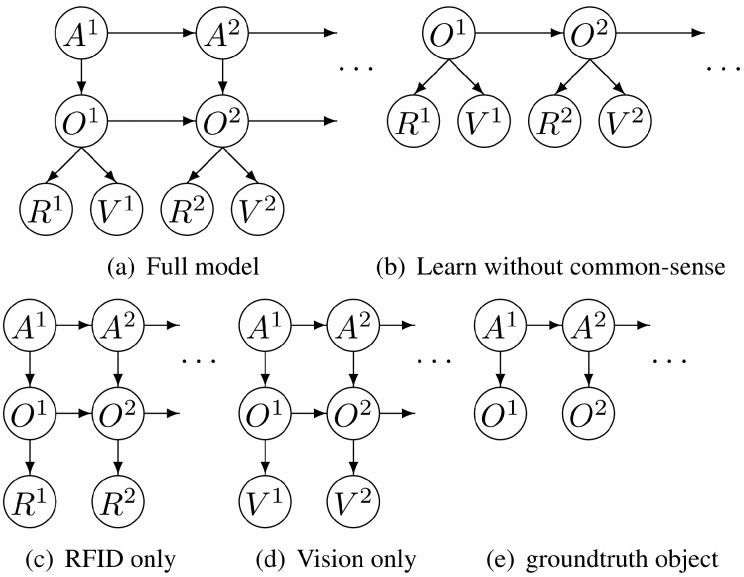
Various graphical models for activity and object recognition in which A, O, R and V represent activity, object, RFID and video frame, respectively (reprinted from [42]).

DBNs have been combined with common sense knowledge in [42]. One of the problems of object-based approaches for activity recognition is that its scalability where a large number of objects must be discriminated, and obtaining labelled training data for each object is difficult. Wu *et al*. proposed using a DBN that has been trained to combine common-sense activity knowledge to tackle this problem. In this work, only information about which objects should be involved was used. For example, making coffee requires the presence of coffee and a cup. This work focused on activities in indoor environments, such as a kitchen or an office. They achieved high activity recognition of more than 80% on their dataset consisting of 16 activities involving 33 objects with significant background clutter. Figure 12 shows their proposed dynamic Bayesian network scheme.

AdaBoost has also been used at the activity level. Fathi *et al*. [8] used AdaBoost with 10 iterations on the histogram of action frequencies for each sequence to recognize seven ADLs performed in a kitchen. The system was able to recognize six out of seven activities correctly and obtained a frame classification accuracy of 45%. A multi-class AdaBoost was used in [46] on spatio-temporal pyramids among a randomized pool of candidate partitions to create a stronger classifier, achieving a classification accuracy of 38.7% on the ADL dataset.

SVM is also used at the activity level. Particularly, the method in [75] used SVM to train a classifier to recognize activities, such as meeting people and eating, from egocentric videos captured with a Microsoft SenseCam. Their dataset was composed of 87,850 images for training and testing, and the system achieved an average F1-measure of 65% on 22 activities. Besides, a linear SVM was used in [11] and [14] on the ADL dataset with 18 daily indoor activities, like making coffee. In [11], the system achieved a 32.6% activity classification accuracy trained with all of the objects and 40.6% accuracy trained with only active objects. With idealized perfect active object detectors, the performance was increased to 77%. A similar system [14], representing activities as sequences of active objects and places, improved performance to 43.3%.

So far, a number of approaches has been proposed to recognize ADLs using egocentric vision. Taking advantage of the wearable camera, they have exploited the constant visibility of hands and the detection of task-relevant objects to describe activities. Multiple methods, including bags of objects or actions and temporal pyramids, have been investigated to represent activities that could then be classified using machine learning approaches, such as SVM, HMM and DBN. However, ADLs recognition is still far from being solved due to current systems’ limitations in unconstrained environments where activities can be performed in different ways, with different subjects and in different contexts.

## 5. Relevant Datasets

During the last few years, there has been an increase in the availability of datasets on egocentric vision due to the affordability of wearable cameras and the associated increase in research in the area. However, compared to other areas, there are not many datasets focused on the recognition of ADLs with egocentric vision due to the privacy problem of these wearable systems. These datasets were collected in two types of scenarios:

- Constrained scenarios: the subjects execute a set of activities in the same environment with the same objects involved, e.g., in a lab setting.
- Unconstrained scenarios: the subjects perform activities in different environments, and objects of the same category, but different appearance are involved, e.g., at home.

The Activities of Daily Living (ADL) dataset has been so far the most complete dataset with one million frames of dozens of people performing unscripted everyday activities, such as drinking water and combing hair, in unconstrained environments [11]. Another widely-used dataset is the Georgia Tech Egocentric Activities (GTEA) dataset, which contains seven types of daily activities performed by four different subjects. Moreover, activities performed in the kitchen have been recorded in several datasets, such as the Carnegie Mellon University Multi-Modal Activity Database (CMU-MMAC), the Georgia Tech Egocentric Activities Gaze+ (GTEA Gaze+) and EDSH-kitchen. Besides activities recorded at home, outdoor activities have also been captured in the The Univ. of Texas at Austin Egocentric (UT Ego) Dataset, LENA and The Hebrew University of Jerusalem (HUJI) EgoSeg Dataset. These videos capture a variety of activities, such as eating, shopping, attending a lecture, driving and cooking, in various scenarios. An eye tracker, which provides a quite accurate eye gaze estimation, was used in the GTEA Gaze+ to support activity recognition. Interactions, such as hand shake, are captured in the first-person social interactions, the Jet Propulsion Laboratory (JPL) First-person Interaction and National University of Singapore (NUS) First-person Interaction Datasets. Table 2 shows the details of interesting datasets from a first-person perspective, including datasets for object and hand detection.

**Table 2:** Datasets for activity recognition in egocentric vision.

**Name**	**Description**	**URL**	**Citations** ${}^{1}$
Activities of Daily Living (ADL) [11]	Unconstrained: A dataset of 1 million frames of dozens of people performing unscripted, everyday activities. The dataset is annotated with activities, object tracks, hand positions and interaction events.	http://people.csail.mit.edu/hpirsiav/codes/ADLdataset/adl.html	143
The University of Texas at Austin Egocentric (UT Ego) Dataset [80]	Unconstrained: The UT Ego Dataset contains 4 videos captured from head-mounted cameras. Each video is about 3–5 h long, captured in a natural, uncontrolled setting. They used the Looxcie wearable camera, which captures video at 15 fps at 320 × 480 resolution. The videos capture a variety of daily activities.	http://vision.cs.utexas.edu/projects/egocentric/index.html	134
First-person social interactions dataset [81]	Unconstrained: This dataset contains day-long videos of 8 subjects spending their day at Disney World Resort in Orlando, Florida. The cameras are mounted on a cap worn by subjects. Elanannotations for the number of active participants in the scene and the type of activity: walking, waiting, gathering, sitting, buying something, eating, *etc.*	http://ai.stanford.edu/~alireza/Disney/	100
Carnegie Mellon University Multi-Modal Activity Database (CMU-MMAC) [67]	Constrained: Multimodal dataset of 18 subjects cooking 5 different recipes (brownies, pizza, *etc.*) containing visual, audio, body motion capture and IMU data. Each frame is labelled with an action, such as take oil or crack egg.	http://kitchen.cs.cmu.edu/	83
Georgia Tech Egocentric Activities (GTEA) [18]	Constrained: This dataset contains 7 types of daily activities, each performed by 4 different subjects. The camera is mounted on a cap worn by the subject.	http://ai.stanford.edu/~alireza/GTEA/	74
Georgia Tech Egocentric Activities-Gaze+ [13]	Constrained: This dataset consists of 7 meal preparation activities collected using eye-tracking glasses, each performed by 10 subjects. Subjects perform the activities based on the given cooking recipes.	http://ai.stanford.edu/~alireza/GTEA_Gaze_Website/	63
EDSH-kitchen [50]	Unconstrained: A video was taken in a kitchenette area while making tea.	https://www.youtube.com/watch?v=N756YmLpZyY	58
Zoombie Dataset [50]	Unconstrained: This dataset consists of three ego-centric videos containing indoor and outdoor scenes where hands are purposefully extended outwards to capture the change in skin colour.	http://www.cs.cmu.edu/~kkitani/datasets/	58
Jet Propulsion Laboratory (JPL) First-person Interaction Dataset [69]	Constrained: This dataset is composed of human activity videos taken from a first-person viewpoint. The dataset particularly aims to provide first-person videos of interaction-level activities, recording how things visually look from the perspective (*i.e.*, viewpoint) of a person/robot participating in such physical interactions.	http://michaelryoo.com/jpl-interaction.html	46
**Name**	**Description**	**URL**	**Citations** ${}^{2}$
Intel 42 Egocentric Objects dataset [38]	Unconstrained: This is a dataset for the recognition of handled objects using a wearable camera. It includes ten video sequences from two human subjects manipulating 42 everyday object instances.	Not currently available	33
The Hebrew University of Jerusalem (HUJI) EgoSeg Dataset [82]	Unconstrained: This dataset consists of 29 videos captured from an ego-centric camera annotated in Elan format. The videos prefixed with “youtube*” were downloaded from YouTube; the rest of the videos were taken by the Hebrew University of Jerusalem researchers and contain various daily activities.	http://www.vision.huji.ac.il/egoseg/videos/dataset.html	18
National University of Singapore (NUS) First-person Interaction Dataset [70]	Unconstrained: 260 videos including 8 interactions in 2 perspectives, third-person and first-person) to create a total of 16 action classes, such as handshake and open doors, captured by a GoPro Camera	https://sites.google.com/site/sanathn/Datasets	5
LENA [74]	Unconstrained: This Google Glass life-logging dataset contains 13 distinct activities performed by 10 different subjects. Each subject recorded 2 clips for one activity. Therefore, each activity category has 20 clips. Each clip takes exactly 30 seconds. Their set of activities are: watching videos, reading, using the Internet, walking straightly, walking back and forth, running, eating, walking up and downstairs, talking on the phone, talking to people, writing, drinking and housework.	http://people.sutd.edu.sg/~1000892/dataset	2

${}^{1}$ Obtained from Google Scholar on 20 October 2015.

## 6. Discussion and Conclusions

As the recognition of ADLs in egocentric vision is an emerging research field, not many approaches have been proposed to address this problem thoroughly. So far, researchers found that the recognition of ADLs with egocentric vision is mainly driven by the objects, particularly those active objects in the scene. Therefore, several object-based approaches have been proposed. However, those methods have a challenge with object recognition due to their intra-class variations in unconstrained scenarios. Current systems are quite far from producing reasonable accuracy, *i.e.*, all of them obtained less than 50%. Moreover, the performance of these systems decreases significantly when applied in an unconstrained environment, *i.e.*, where the evaluation phase and training phase are not performed under the same conditions. However, with the recent development of convolutional neural networks, which have been applied successfully for object recognition in third-person vision in unconstrained environments [83,84,85], it is expected that their usage within egocentric vision will lead to significant improvement in object-driven ADL recognition.

Wearable cameras bring not only advantages, but also challenges for recognizing ADLs. While objects and hands are almost always visible to cameras, which is beneficial for recognition, motion blur, hand occlusion and background clutter are still far from being solved; even if background clutter, in some extent, has been tackled by the estimation of the area of interest using eye tracking, foreground subtraction or saliency maps. In the meanwhile, the benefits of egocentric vision have been exploited and achieved initial results. However, only coarse descriptions of the scene based on objects and hands have been analysed. Another challenge is the privacy issues, which make it difficult to record activities from people at home. As a result, there are only a few datasets recorded for the training phase, which contributes to limited classification accuracy.

In the future, research opportunities are still open. There are several directions that should be considered:

- Current DBN systems for activity recognition, which exploit the order in which the actions take place, are sensitive to noise, as there could be not only one, but multiple orders in which an activity can be completed. Therefore, another approach that takes into account alternative orders in which actions occur within an activity should be investigated; and
- The problem of intra-class variation in object recognition in an unconstrained environment can be tackled by bio-inspired algorithms motivated by the idea that an object with its variations shares the same characteristics.

This review provides a summary of the state of the art from the research point of view about the methods that have been applied to recognize ADLs in egocentric settings. The approaches of computer vision and machine learning have been presented and discussed to provide an overview of what has been done so far.
